# Trends in neonatal mortality on the first day of life in Japan, Korea, and Taiwan

**DOI:** 10.1186/s12889-025-23867-x

**Published:** 2025-10-03

**Authors:** Fu-Wen Liang, Chia-Sui Chou, Hsiu-Lin Chen, Mei-Jy Jeng, Hung-Chieh Chou, Ming-Chou Chiang, Reyin Lien, Hung-Yang Chang, Chun-Chih Peng, Tsung-Hsueh Lu

**Affiliations:** 1 Department of Public Health, College of Health Sciences, Kaohsiung Medical University, Kaohsiung, Taiwan; 2 Department of Medical Research, Kaohsiung Medical University Hospital, Kaohsiung, Taiwan; 3 Center for Big Data Research, Kaohsiung Medical University, Kaohsiung, Taiwan; 4 Neonatal Medical Care Center, Department of Pediatrics, Taipei Veterans General Hospital, Taipei, Taiwan; 5 Institute of Emergency and Critical Care Medicine, School of Medicine, National Yang Ming Chiao Tung University, Taipei, Taiwan; 6 Department of Pediatrics, School of Medicine, National Yang Ming Chiao Tung University, Taipei, Taiwan; 7 Department of Pediatrics, Kaohsiung Medical University Hospital, Kaohsiung, Taiwan; 8 Department of Respiratory Therapy, College of Medicine, Kaohsiung Medical University, Kaohsiung, Taiwan; 9 School of Medicine, College of Medicine, National Sun Yat-sen University, Kaohsiung, Taiwan; 10 Neonatology Division, Department of Pediatrics, National Taiwan University Children’s Hospital, Taipen, Taiwan; 11 Division of Neonatology, Department of Pediatrics, Chang Gung Memorial Hospital, Linkou Tao-Yuan, Taiwan; 12 Department of Pediatrics, School of Medicine, Chang Gung University, Tao-Yuan, Taiwan; 13 Division of Neonatology, Department of Pediatrics, MacKay Children’s Hospital, Taipen, Taiwan; 14 Department of Medicine, MacKay Medical University, New Taipei, Taiwan; 15 Department of Public Health, College of Medicine, National Cheng Kung University, Tainan, Taiwan

**Keywords:** Birth certificate, International comparisons, Mortality trends, Neonatal mortality rates, Vital statistics

## Abstract

**Background:**

Studies have indicated that the risk of death on the first day of life (day 0) was higher than risk of death during other periods (days 1 to 6 and 7 to 27). However, little is known about whether the pattern of mortality trends on day 0 differs from those on days 1 to 6 and 7 to 27. We aimed in this study to examine NMRs trends by age at death in Japan, Korea, and Taiwan.

**Methods:**

In this cross-sectional study, we calculated NMRs (deaths per 1000 live births) by age at death from 2005 to 2021 in Japan, 2005 to 2022 in Korea, and 2005 to 2023 in Taiwan. Joinpoint regression model was used to estimate the annual percent change (APC) for each segment of the trend in NMRs to examine whether the trend changed significantly.

**Results:**

A slowdown of decreasing trend on days 0 to 27 was observed from 2015 to 2021 with APC of − 4.3% to − 1.5% in Japan and from 2008 to 2018 with APC of − 8.5% to − 1.4% in Korea. In contrast, an initial decline followed by an increase pattern of trend was noted in Taiwan with APC of − 2.5% from 2005 to 2014 to 2.1% from 2014 to 2023. In Japan, the slowdown was mainly due to the levelling-off in the decline in NMRs for days 1 to 6. In Korea, the slowdown was mainly attributed to the levelling-off in the decline in NMRs for days 7 to 27. In Taiwan, the prominent change was primarily due to the changes in day 0 NMRs.

**Conclusions:**

Further analyses are needed to explore potential factors associated with the particular pattern of trends of NMRs at specific age-at-death group. Neonatal mortality on the first day of life is not an appropriate indicator of neonatal care quality, as it may be influenced by artifacts related to birth certification practices.

## Background

Neonatal mortality rate (NMR) is defined as the number of deaths during the first 28 days of life per 1000 live births and a sensitive marker of a health system’s response to its most vulnerable citizens [1, 2]. NMR has been recognized as a target (3.2.2) of the United Nations’ Sustainable Development Goal, which aims to end preventable neonatal deaths by 2030, with all countries aiming to reduce NMR to at least as low as 12 deaths per 1000 live births [3]. According to UN Inter-agency Group for Child Mortality Estimation (IGME), the NMR was 19 deaths per 1000 live births in 2000 and declined to 6 deaths per 1000 live births in 2023 in West Pacific region [4]. However, levelling-off or rising NMRs have been reported in some European high-income countries [5–7]. For example, the infant mortality rate (IMR) in England was 3.5 deaths per 1000 live births in 2014 and increased to 3.7 deaths per 1000 live births in 2016, driven by a rise in early neonatal deaths (days 0 to 6) [5]. A study by Trinh et al. indicated an increase in IMR in France beginning in 2012, with a slope of 0.0033, also attributed to an increase in early neonatal mortality. Their study further suggested that the proportion of deaths occurring on the first day of life (day 0) rose from 24% in 2001 to 26% in 2019 [6]. Studies have shown that the risk of death on day 0 was higher than risk of death during other periods (days 1 to 6 and 7 to 27) [8–12]. However, little is known about whether the pattern of mortality trends on day 0 differs from those on days 1 to 6 and 7 to 27. We aimed in this study to examine NMRs trends by age at death in Japan, Korea, and Taiwan. These three countries were selected for comparison due to their geographic proximity, historical connectedness, similar cultural context, and the availability of high-quality mortality data.

## Methods

In this cross-sectional study, mortality data for Japan and Korea were extracted from the World Health Organization mortality database [13] and data for Taiwan were obtained from the Taiwan Cause of Death Statistics Database [14]. The study periods covered the years 2005 to 2021 for Japan, 2005 to 2022 for Korea, and 2005 to 2023 for Taiwan. The NMR was calculated for 4 age-at-death periods: days 0, 1 to 6, 7 to 27, and 0 to 27. The proportions of days 0, 1 to 6, 7 to 27 deaths among total neonatal deaths (days 0 to 27) in each year in each country were computed for comparisons with previous studies. Joinpoint regression software (version 5.0; National Cancer Institute) was used to estimate the annual percent change (APC) and 95% confidence intervals (CIs) for each segment of the trend of NMRs to examine whether the trend changed significantly [15].

## Results

During the study period, a total of 17 856, 12 443, and 9140 neonatal deaths (days 0 to 27) occurred in Japan (2005 through 2021), Korea (2005 through 2022), and Taiwan (2005 through 2023), respectively, with corresponding NMR of 1.05, 1.73, and 2.56 deaths per 1000 live births, respectively. The yearly number of deaths, NMRs, and proportions of day 0 deaths by age at death periods for Japan, Korea, and Taiwan are illustrated in Tables 1 and 2, and Table 3, respectively. Japan consistently had the lowest overall NMRs for days 1 to 6, 7 to 27, and 0 to 27 compared to Korea and Taiwan. Korea exhibited the lowest overall NMRs for day 0 compared to Japan and Taiwan, with rates of 0.52, 0.43, and 1.01 deaths per 1000 live births, respectively. On the contrary, Japan consistently had the highest proportion of day 0 deaths among total 0 to 27 neonatal deaths compared to Korea and Taiwan in each study year, with an overall proportion of 49% in Japan, 25% in Korea, and 39% in Taiwan (Tables 1 and 2, and 3).

**Table 1:** Number of deaths (No), mortality rates (deaths per 1000 live births) and proportions (%) of neonatal deaths by age at death in Japan from 2005 to 2021

	Day 0			1–6 days			7–27 days			0–27 days		
Year	No	Rate	%	No	Rate	%	No	Rate	%	No	Rate	%
2005	691	0.65	46	400	0.38	26	419	0.39	28	1510	1.42	100
2006	644	0.59	45	409	0.37	28	391	0.36	27	1444	1.32	100
2007	673	0.62	47	379	0.35	26	382	0.35	27	1434	1.32	100
2008	640	0.59	48	329	0.30	25	362	0.33	27	1331	1.22	100
2009	583	0.54	46	291	0.27	23	380	0.36	30	1254	1.17	100
2010	592	0.55	51	286	0.27	25	289	0.27	25	1167	1.09	100
2011	549	0.52	48	275	0.26	24	323	0.31	28	1147	1.09	100
2012	565	0.54	53	225	0.22	21	275	0.27	26	1065	1.03	100
2013	506	0.49	49	246	0.24	24	274	0.27	27	1026	1.00	100
2014	476	0.47	50	235	0.23	25	241	0.24	25	952	0.95	100
2015	452	0.45	50	213	0.21	24	237	0.24	26	902	0.90	100
2016	441	0.45	50	235	0.24	27	198	0.20	23	874	0.89	100
2017	447	0.47	54	178	0.19	21	207	0.22	25	832	0.88	100
2018	424	0.46	53	190	0.21	24	187	0.20	23	801	0.87	100
2019	401	0.46	53	177	0.20	23	177	0.20	23	755	0.87	100
2020	377	0.45	54	175	0.21	25	152	0.18	22	704	0.84	100
2021	337	0.42	51	169	0.21	26	152	0.19	23	658	0.81	100
Overall	8798	0.52	49	4412	0.26	25	4646	0.27	26	17,856	1.05	100

**Table 2:** Number of deaths (No), mortality rates (deaths per 1000 live births) and proportions (%) of neonatal deaths by age at death in Korea from 2005 to 2022

	Day 0			1–6 days			7–27 days			0–27 days		
Year	No	Rate	%	No	Rate	%	No	Rate	%	No	Rate	%
2005	257	0.59	25	438	1.01	43	334	0.77	32	1029	2.37	100
2006	214	0.48	22	401	0.89	41	363	0.81	37	978	2.18	100
2007	230	0.47	24	403	0.82	42	335	0.68	35	968	1.96	100
2008	209	0.45	24	402	0.86	47	247	0.53	29	858	1.84	100
2009	204	0.46	27	317	0.71	41	246	0.55	32	767	1.72	100
2010	226	0.48	26	375	0.80	44	253	0.54	30	854	1.82	100
2011	216	0.46	27	321	0.68	40	269	0.57	33	806	1.71	100
2012	208	0.43	25	350	0.72	42	276	0.57	33	834	1.72	100
2013	177	0.41	24	321	0.74	43	254	0.58	34	752	1.72	100
2014	174	0.40	23	294	0.68	40	275	0.63	37	743	1.71	100
2015	189	0.43	28	266	0.61	39	222	0.51	33	677	1.54	100
2016	165	0.41	25	258	0.64	39	237	0.58	36	660	1.62	100
2017	145	0.41	27	202	0.56	37	199	0.56	36	546	1.53	100
2018	143	0.44	27	202	0.62	38	188	0.58	35	533	1.63	100
2019	120	0.40	26	192	0.63	41	155	0.51	33	467	1.54	100
2020	87	0.32	25	125	0.46	36	133	0.49	39	345	1.27	100
2021	94	0.36	29	129	0.50	40	103	0.40	32	326	1.25	100
2022	60	0.24	20	145	0.58	48	95	0.38	32	300	1.20	100
Overall	3118	0.43	25	5141	0.71	41	4184	0.58	34	12,443	1.73	100

**Table 3:** Number of deaths (No), mortality rates (deaths per 1000 live births) and proportions (%) of neonatal deaths by age at death in Taiwan from 2005 to 2023

	Day 0			1–6 days			7–27 days			0–27 days		
Year	No	Rate	%	No	Rate	%	No	Rate	%	No	Rate	%
2005	214	1.04	35	235	1.14	39	156	0.76	26	605	2.93	100
2006	178	0.87	32	237	1.15	43	139	0.68	25	554	2.69	100
2007	215	1.06	37	224	1.10	38	149	0.73	25	588	2.89	100
2008	179	0.91	33	221	1.12	41	138	0.70	26	538	2.74	100
2009	157	0.82	35	175	0.91	39	120	0.62	27	452	2.35	100
2010	153	0.92	36	178	1.07	41	98	0.59	23	429	2.58	100
2011	207	1.04	39	193	0.97	36	130	0.66	25	530	2.67	100
2012	184	0.78	34	219	0.93	41	135	0.58	25	538	2.29	100
2013	145	0.74	32	209	1.07	46	105	0.54	23	459	2.35	100
2014	164	0.78	36	181	0.86	40	113	0.53	25	458	2.17	100
2015	214	1.00	40	210	0.99	39	115	0.54	21	539	2.53	100
2016	205	0.99	41	195	0.94	39	105	0.51	21	505	2.43	100
2017	216	1.11	44	150	0.77	31	120	0.62	25	486	2.50	100
2018	205	1.13	43	177	0.98	37	92	0.51	19	474	2.62	100
2019	197	1.13	48	136	0.78	33	80	0.46	19	413	2.36	100
2020	173	1.07	45	134	0.83	35	80	0.50	21	387	2.40	100
2021	203	1.29	48	129	0.82	30	93	0.59	22	425	2.71	100
2022	192	1.40	50	134	0.98	35	57	0.41	15	383	2.79	100
2023	198	1.48	53	114	0.85	30	65	0.49	17	377	2.82	100
Overall	3599	1.01	39	3451	0.97	38	2090	0.59	23	9140	2.56	100

**Fig. 1:**
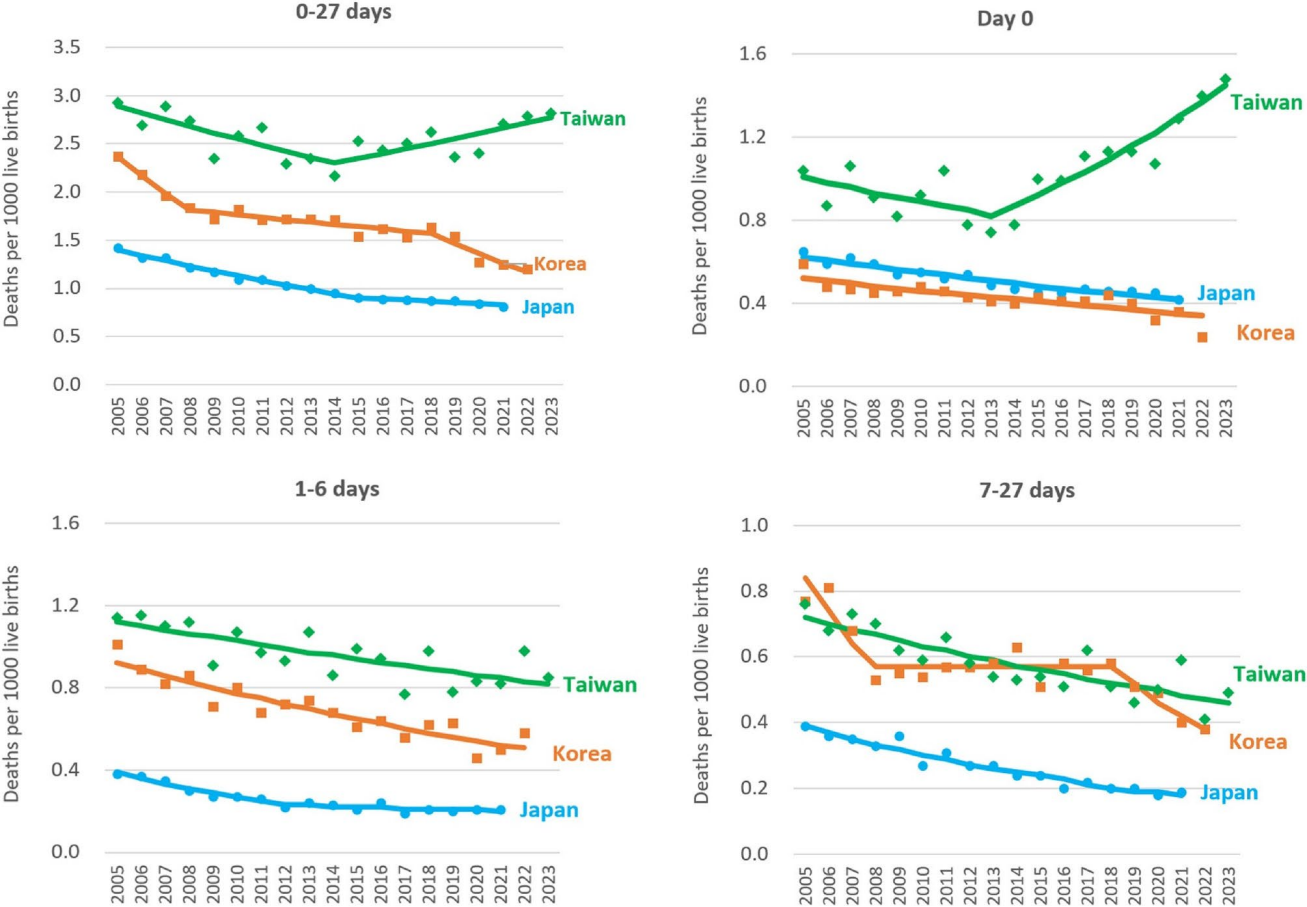
Trends in neonatal mortality rates (deaths per 1000 live births) by age at death in Japan, Korea, and Taiwan (The dots indicate the observed mortality rates, and the solid lines indicate the modelled rates based on the results of joinpoint regression model.)

The mortality trends for different age at death periods in the three countries are illustrated in Fig. 1, and the results of the joinpoint regression model are presented in Table 4. A slowdown of decreasing trend in 0 to 27 days NMRs was observed from 2015 to 2021 with APC of − 4.3% to − 1.5% in Japan and from 2008 to 2018 with APC of − 8.5% to − 1.4% in Korea. In contrast, an initial decline followed by an increase pattern of trend was noted in Taiwan with APC of − 2.5% from 2005 to 2014 to 2.1% from 2014 to 2023.

**Table 4:** Annual percentage changes (APC) and 95% confidence intervals (CIs) for the neonatal mortality rates by age of death in japan, korea, and Taiwan

	Japan			Korea			Taiwan		
	Year	APC	(95% CI)	Year	APC	(95% CI)	Year	APC	(95% CI)
0 day									
Trend 1	2005 to 2021	−2.5%	(−2.9 to −2.1)***	2005 to 2022	−2.5%	(−3.4 to −1.6)***	2005 to 2013	−2.5%	(−5.4 to 0.6)
Trend 2							2013 to 2023	5.8%	(3.5 to 8.2)***
1 to 6 days									
Trend 1	2005 to 2012	−7.1%	(−9 to −5.1)***	2005 to 2022	−3.5%	(−4.2 to −2.7)***	2005 to 2023	−1.8%	(−2.5 to −1)***
Trend 2	2012 to 2021	−1.5%	(−3.3 to 0.3)						
7 to 27 days									
Trend 1	2005 to 2021	−4.8%	(−5.4 to −4.2)***	2005 to 2008	−12.3%	(−19.9 to −4)**	2005 to 2023	−2.4%	(−3.2 to −1.7)***
Trend 2				2008 to 2018	0.1%	(−1.8 to 2.1)			
Trend 3				2018 to 2022	−10%	(−18.1 to −0.9)*			
0 to 27 days									
Trend 1	2005 to 2015	−4.3%	(−4.7 to −3.9)***	2005 to 2008	−8.5%	(−12.8 to −4.1)**	2005 to 2014	−2.5%	(−3.9 to −1.0)**
Trend 2	2015 to 2021	−1.5%	(−2.5 to −0.4)*	2008 to 2018	−1.4%	(−2.5 to −0.4)*	2014 to 2023	2.1%	(0.5 to 3.7)*
Trend 3				2018 to 2022	−7.0%	(−11.5 to −2.3)**			

***P value < 0.001; **P value < 0.01; *P value < 0.05

The APCs and 95% CI were estimated based on the joinpoint regression program

In Japan, the slowdown was mainly due to the levelling-off in the decline in NMRs for days 1 to 6, with the APC changing from − 7.1% during the period between 2005 and 2012 to − 1.5% during the period between 2012 and 2021. In Korea, the slowdown was mainly attributed to the levelling-off in the decline in NMRs for days 7 to 27, with the APC changing from − 12.3% during the period between 2005 and 2008 to 0.1% during the period between 2008 and 2018. In Taiwan, the prominent change was primarily due to the changes in day 0 NMRs, with the APC of − 2.5% during the period between 2005 and 2014 and changing to 5.8% increase during the period between 2014 and 2022.

## Discussion

The findings of this study reveal that among three high income West Pacific Asian countries, Japan consistently had the lowest NMRs for days 1 to 6, 7 to 27, and 0 to 27, while Korea exhibited the lowest NMRs for day 0 in each study year. Regarding the pattern of mortality trends, the declines in NMRs slowed down in Japan and Korea were driven by trends in mortality in different age at death periods in each country. Conversely, a decline followed by an increase in the NMRs was noted in Taiwan, with the increase mainly attributed to rising in day 0 mortality from 2014 to 2023.

The slowdown in the declines of NMRs in Japan and Korea, along with an increase in NMRs in Taiwan, mirrors similar trends observed in some high-income European countries [5–7]. For instance, in the United Kingdom, the APC in the early (day 0 to 6) NMR was − 0.8% for the period between 2008 and 2014 and 0.9% for that between 2014 and 2016 [5]. In England and Wales, the trends in early NMRs were primarily driven by increases in deaths on day 0 from 1.33 deaths per 1000 live births in 2014 to 1.60 deaths per 1000 live births in 2017 [5]. Similarly, in France, early NMRs increased from 2012 to 2019 [7]. In both cases, the increase in early NMRs was mainly due to an increase in the proportions of newborns at < 24 gestational weeks [5, 6].

Before exploring the traditional maternal, fetal, quality of health care and delivery system factors associated with NMRs during the antenatal, perinatal, and postneonatal periods, we should first consider possible artefact bias. One possible explanation for the abrupt increase in Taiwan’s day 0 NMRs since 2014 was the changes in obstetricians’ behavior regarding the registration of live births for periviable newborns [16]. Table 5 illustrates the proportions (%) of newborns with a gestational age < 22 weeks and birthweight < 500 g (a proxy measure of periviable newborns) among live births from 2004 to 2019 [17]. The proportion of birthweight < 500 g increased dramatically from 0.035% in 2014 to 0.064% in 2015.

**Table 5:** Numbers and proportions of live births with gestational age (GA) < 22 weeks and birthweight (BW) < 500 g in Taiwan from 2009 to 2020

Year	Live births	GA < 22 wks	%	BW < 500 g	%
2009	192,465	32	0.017	56	0.029
2010	166,630	34	0.020	59	0.035
2011	198,387	52	0.026	88	0.044
2012	234,575	53	0.023	82	0.035
2013	195,251	30	0.015	76	0.039
2014	211,734	43	0.020	74	0.035
2015	213,714	69	0.032	136	0.064
2016	207,837	77	0.037	121	0.058
2017	195,115	91	0.047	151	0.077
2018	181,084	72	0.040	119	0.066
2019	176,006	90	0.051	145	0.082
2020	162,455	72	0.044	105	0.065

Source: Taiwan Health Promotion Administration. Annual Report of Birth Reporting System. Available at

https://www.hpa.gov.tw/Pages/TopicList.aspx?nodeid=649 Accessed at May 24, 2025

The changes in obstetricians’ behaviors regarding the registration of live births for periviable newborns were mainly influenced by the increase in childbirth subsidies offered by local governments to address the low birth rate issue in Taiwan. Some families of pregnant women who underwent late pregnancy terminations requested obstetricians to issue birth certificates. This request aimed to enable them to claim childbirth subsidies, especially in cases where fetuses extracted had weak heartbeats and died immediately afterward. In such cases, a death certificate would be issued after civil registration. Some obstetricians were willing to issue a birth certificate followed by a death certificate, while others were not, leading to disputes in clinical settings [16]. In July 2015, a legal consultant letter published in the Taiwan Association of Obstetrics and Gynecology newsletter in favor of issuing birth certificates for all fetuses with gestational ages of > 20 weeks with heartbeats [18]. Subsequently, some hospitals set guidelines based on this letter, resulting in a notable increase in the number of day 0 deaths from 95 cases between January and June to 115 cases between July and December in 2015, with a persistent increase thereafter [16].

Joseph et al. indicated that an increase in stillbirth rates in Canada between 2000 and 2010 was primarily attributed to an increase in late pregnancy terminations [19]. Studies conducted in the United States and the United Kingdom have likewise demonstrated geographic variations in the reporting of live births and day 0 deaths for periviable newborns, thereby reducing the comparability of NMRs across regions [20–23]. Similarly, the increase in issuing birth certificates and death certificates for some artificial abortions since 2015 in Taiwan was limited to some obstetricians in certain regions [24].

Regarding the proportion of day 0 deaths among all neonatal deaths, Oza et al. highlighted significant variations in this proportion across 31 industrialized nations. The highest proportion of day 0 deaths was noted in Switzerland (71%), followed by Canada (69%), and Austria (62%). In contrast, the lowest proportions, ranking in the top three, were observed in Czechia (23%), Cyprus (27%), and Luxembourg (29%) [9]. Compared to the aforementioned countries, the proportions in Japan and Taiwan were moderate, while that in Korea was low. One possible explanation for the variations in the proportion of day 0 deaths across countries is differences in certification practices regarding whether periviable newborns are classified as stillbirths or live births [25–28]. Caution is warranted when comparing the proportion of day 0 deaths between countries. A high proportion of day 0 deaths in one country may simply reflect a relatively low number of deaths on days 1–6 or 7–27, rather than a true difference in early neonatal mortality. These proportions can be influenced by certification practices and the overall distribution of deaths throughout the neonatal period. Therefore, both the NMR and the proportion of day 0 deaths are not reliable indicators of the quality of neonatal care.

This study has several limitations. First, we used the official published vital statistics data, which did not include information on gestational age and birthweight. As a result, we were unable to analyze NMRs by age at death with a minimum threshold of 22 weeks of gestational age and 500 gm birthweight across the three countries, which could provide information on possible effects on certification practice variations on registration of live births for periviable newborns. In OECD statistics, two NMRs, with and without a minimum threshold, are presented for member countries [29]. Second, the data did not permit detailed analysis of the timing of death in hours or minutes for day 0 deaths across the three countries. For example, one newborn might be delivered at 11:00 PM and die 30 min later, while another might be born at 2:00 AM and survive for 20 h. Both cases would be recorded as deaths occurring on the first day of life, despite the substantial difference in survival time. Third, the absence of data regarding several mortality risk factors, such as maternal age, education level, comorbidities during pregnancy, and antenatal checkups, precluded further analysis.

Through the international comparisons of NMRs trends in three high-income West Pacific Asian countries, we could identify problems in particular country. The findings of this study revealed a deceleration in the decline of NMRs in Japan and Korea, whereas in Taiwan, there was a decline followed by an increase in the NMR. The different patterns of changes in the NMRs in three countries were driven by mortality trends across periods after birth: 1 to 6 days in Japan, 7 to 27 days in Korea, and 0 days in Taiwan. Further analyses are necessary to explore potential factors associated with the levelling and increasing mortality rates in particular age-at-death group. In conclusion, neonatal mortality on the first day of life is not an appropriate indicator of neonatal care quality, as it can be influenced by artifacts related to birth certification practices.

## Data Availability

Data is provided within the manuscript.

## References

[1] World Health Organization. 2018 Global Reference List of 100 Core Health Indicators (plus Health-Related SDGs). Geneva: World Health Organization, 2018. Available at: https://iris.who.int/handle/10665/259951 Accessed at September 24, 2023.

[2] WHO/UNICEF. Every Newborn: An Action Plan to End Preventable Deaths. Geneva: World Health Organization. 2014. https://www.who.int/initiatives/every-newborn-action-plan Accessed Aug 10, 2023.

[3] United Nation, Department of Economic and Social Affair, Statistics Division. The Sustainable Development Goals Indicators Metadata Repository. Available at: https://unstats.un.org/sdgs/metadata/ Accessed Aug 10, 2023.

[4] United Nations Inter-agency Group for Child Mortality Estimation (UN IGME). Levels & trends in child mortality: report 2024. New York: United Nations Children’s Fund; 2025.

[5] Nath S, Hardelid P, Zylbersztejn A. Are infant mortality rates increasing in England?? The effect of extreme prematurity and early neonatal deaths. J Public Health (Oxf). 2021;43(3):541–50. 32119086 10.1093/pubmed/fdaa025 PMC8458015

[6] Trinh NT, de Visme S, Cohen JF, et al. Recent historic increase of infant mortality in france: A time–series analysis, 2001 to 2019. Lancet Reg Health Eur. 2022;16:100339. 35252944 10.1016/j.lanepe.2022.100339 PMC8891691

[7] Onambele L, San-Martin-Rodríguez L, Niu H, et al. Infant mortality in the European Union: a time trend analysis of the 1994–2015 period. Anales de Pediatría (English Edition). 2019;91(4):219–27. 30857913 10.1016/j.anpedi.2018.10.022

[8] Lawn JE, Blencowe H, Oza S, et al. Every newborn: progress, priorities, and potential beyond survival. Lancet. 2014;384:189–205. 24853593 10.1016/S0140-6736(14)60496-7

[9] Oza S, Cousens SN, Lawn JE. Estimation of daily risk of neonatal death, including the day of birth, in 186 countries in 2013: a vital-registration and modelling-based study. Lancet Glob Health. 2014;2: e635–44. 25442688 10.1016/S2214-109X(14)70309-2

[10] Auger N, Bilodeau-Bertrand M, Nuyt AM. Dangers of death on the first day of life by the minute. J Perinatol. 2015;35:958–64. 26334397 10.1038/jp.2015.107

[11] Baqui AH, Mitra DK, Begum N, et al. Neonatal mortality within 24 hours of birth in six low-and lower-middle-income countries. Bull World Health Organ. 2016;94:752–B758. 27843165 10.2471/BLT.15.160945 PMC5043199

[12] Teixeira JA, Araujo WR, Maranhão AG, et al. Mortality on the first day of life: trends, causes of death and avoidability in eight Brazilian federative units, between 2010 and 2015. Epidemiol Serv Saude. 2019;28:e2018132. 30785573 10.5123/S1679-49742019000100006

[13] World Health Organization. WHO mortality database. https://www.who.int/data/data-collection-tools/who-mortality-database Accessed at July 10, 2023.

[14] Ministry of Health and Welfare Taiwan. Cause of death statistics. Accessed https://www.mohw.gov.tw/np-128-2.html Accessed July 10, 2023.

[15] The National Cancer Institute. The Joinpoint Trend Analysis Software. https://surveillance.cancer.gov/joinpoint/ Accessed July 10, 2023.

[16] Liang FW, Lu TH, Chiang TL. Over-registration and increasing neonatal mortality in Taiwan. Taiwan J Public Health. 2023;42:352–9.

[17] Health Promotion Administration, Ministry of Health and Welfare, Taiwan. 2022 Annual Report of Birth Reporting System. Taipei: Health Promotion Administration, 2022. Available: https://www.hpa.gov.tw/Pages/TopicList.aspx?nodeid=649

[18] Kao TF. Do not refer mid-trimester abortion patients to avoid lawbreaking. Bull Taiwan Obstet Gynecol Assoc. 2015;225:38–42.

[19] Joseph KS, Kinniburgh B, Hutcheon JA, et al. Determinants of increases in stillbirth rates from 2000 to 2010. CMAJ. 2013;185:E345–51. 23569166 10.1503/cmaj.121372 PMC3652963

[20] Ehrenthal DB, Wingate MS, Kirby RS. Variation by state in outcomes classification for deliveries less than 500 G in the united States. Matern Child Health J. 2011;15:42–8. 20111990 10.1007/s10995-010-0566-y

[21] Woods CR, Davis DW, Duncan SD, et al. Variation in classification of live birth with newborn period death versus fetal death at the local level may impact reported infant mortality rate. BMC Pediatr. 2014;14:108. 24755366 10.1186/1471-2431-14-108 PMC4000129

[22] Goyal NK, DeFranco E, Kamath-Rayne BD, et al. County-level variation in infant mortality reporting at early previable gestational ages. Paediatr Perinat Epidemiol. 2017;31:385–91. 28722799 10.1111/ppe.12376 PMC6173802

[23] Smith L, Draper ES, Manktelow BN, et al. Comparing regional infant death rates: the influence of preterm births < 24 weeks of gestation. Arch Dis Child Fetal Neonatal Ed. 2013;98:F103–7. 22684158 10.1136/fetalneonatal-2011-301359 PMC3582045

[24] Wang LY, Chang YS, Liang FW, Lin YC, Lin YJ, Lu TH, Lin CH. Comparing regional neonatal mortality rates: the influence of registration of births as live born for birth weight < 500 g in Taiwan. BMJ Paediatr Open. 2019;3:e000526. 31414067 10.1136/bmjpo-2019-000526 PMC6668753

[25] Kramer MS, Platt RW, Yang H, Haglund B, Cnattingius S, Bergsjo P. Registration artifacts in international comparisons of infant mortality. Paediatr Perinat Epidemiol. 2002;16:16–22. 11862950 10.1046/j.1365-3016.2002.00390.x

[26] Joseph KS, Liu S, Rouleau J, et al. Influence of definition based versus pragmatic birth registration on international comparisons of perinatal and infant mortality: population based retrospective study. BMJ. 2012;344:e746. 22344455 10.1136/bmj.e746 PMC3281499

[27] Deb-Rinker P, León JA, Gilbert NL, Rouleau J, Andersen AM, Bjarnadóttir RI, Gissler M, Mortensen LH, Skjærven R, Vollset SE, Zhang X. Differences in perinatal and infant mortality in high-income countries: artifacts of birth registration or evidence of true differences? BMC Pediatr. 2015;15: 112. 26340994 10.1186/s12887-015-0430-8 PMC4560894

[28] Smith LK, Blondel B, Zeitlin J, Euro-Peristat Scientific Committee. Producing valid statistics when legislation, culture and medical practices differ for births at or before the threshold of survival: report of a European workshop. BJOG. 2020;127:314–8. 31580509 10.1111/1471-0528.15971 PMC7003918

[29] Organization of Economic Co-operation and Development (OECD). OECD Statistics: Health Status, 2023. Available at https://stats.oecd.org/index.aspx?queryid=24879 Accessed September 24, 2023.

